# Haemobilia in a patient on oral anticoagulation: A surgical case report

**DOI:** 10.1016/j.ijscr.2025.110853

**Published:** 2025-01-06

**Authors:** Iman Hameed, Yahya Al-Habbal

**Affiliations:** Department of Upper Gastrointestinal/Hepatobiliary Surgery, Western Hospital, Footscray, VIC 3011, Australia

**Keywords:** Haemobilia, Anticoagulation, ERCP, Biliary imaging, Case report

## Abstract

**Introduction:**

Haemobilia causing obstructive jaundice is a rare complication with most occurrences reported post instrumentation e.g. endoscopic retrograde cholangiopancreatography (ERCP), percutaneous transhepatic cholangioagraphy (PTC) and, trans-cystic duct exploration or due to hepatic tree pseudoaneurysms. Traumatic haemobilia typically presents with the classical triad of right upper quadrant pain, jaundice and upper gastrointestinal bleeding. On imaging, an obstructed biliary tree is commonly found dilated.

**Case presentation:**

We report a case of a large obstructing blood clot causing biliary sepsis for a patient on oral anticoagulation. The patient had no classical triad findings or demonstrable evidence of biliary obstruction on imaging. The patient was managed with clot retrieval via ERCP and sphincterotomy; anticoagulant was resumed seven days post procedure.

**Discussion:**

Haemobilia is a rare consequence in patients on anticoagulation therapy. The management principles are coagulopathy correction and obstruction relief. The pathophysiology in patients without bleeding disorders remains unknown.

**Conclusion:**

Although rare, haemobilia can be a cause of obstructive jaundice for a patient on anticoagulation.

## Highlights

• Traumatic haemobilia causing obstructive jaundice is a rare occurrence and requires prompt management.
• Haemobilia typically presents with classical Quincke's triad however this may not always be the case
• Patients with gallstones or biliary stones on anticoagulation are at risk of spontaneous haemobilia

## 1 Introduction

We report on a case about a patient with biliary sepsis secondary to an obstructive blood clot in his distal bile duct who was on anticoagulation therapy. The patient had minimal symptoms and no radiological evidence of bile duct obstruction. We propose haemobilia should be a differential diagnosis for patients on anticoagulation presenting with biliary sepsis despite atypical symptoms and no evidence on imaging.

## 2 Methodology

This case has been reported in line with SCARE [1] and PROCESS Guidelines [2].

## 3 Case description

A 74-year-old comorbid and obese gentleman presented to the emergency department with fever, chest pain, dyspnoea and generalised abdominal pain. The patient's pre-existing medical conditions included pulmonary hypertension, heart failure, chronic kidney disease, chronic obstructive pulmonary disorder and atrial fibrillation, which he was anticoagulated with apixaban for. There was no prior history of biliary instrumentation or abdominal surgery. Examination was challenging due to his large body habitus and BMI of 41. Biochemical investigations revealed cholestatic liver function tests and rising inflammatory markers. These were the following results; Haemoglobin 110 (130–180 g/L), white cell count 8.8 (4.0–11.0 × 10^9^/L), platelet 153 (150–450 × 10^9^/L), lipase 63 (60 units/L), total bilirubin 55 (<20 μmol/L), alkaline phosphatase (ALP) 189 (30–110 U/L), gamma-glutamyl transferase (GGT) 265 (<55 U/L), C-reactive protein (CRP) 182 (<10 mg/L). His coagulation profile was normal, including an international normalised ratio (INR) of 1.0.

**Fig. 1:**
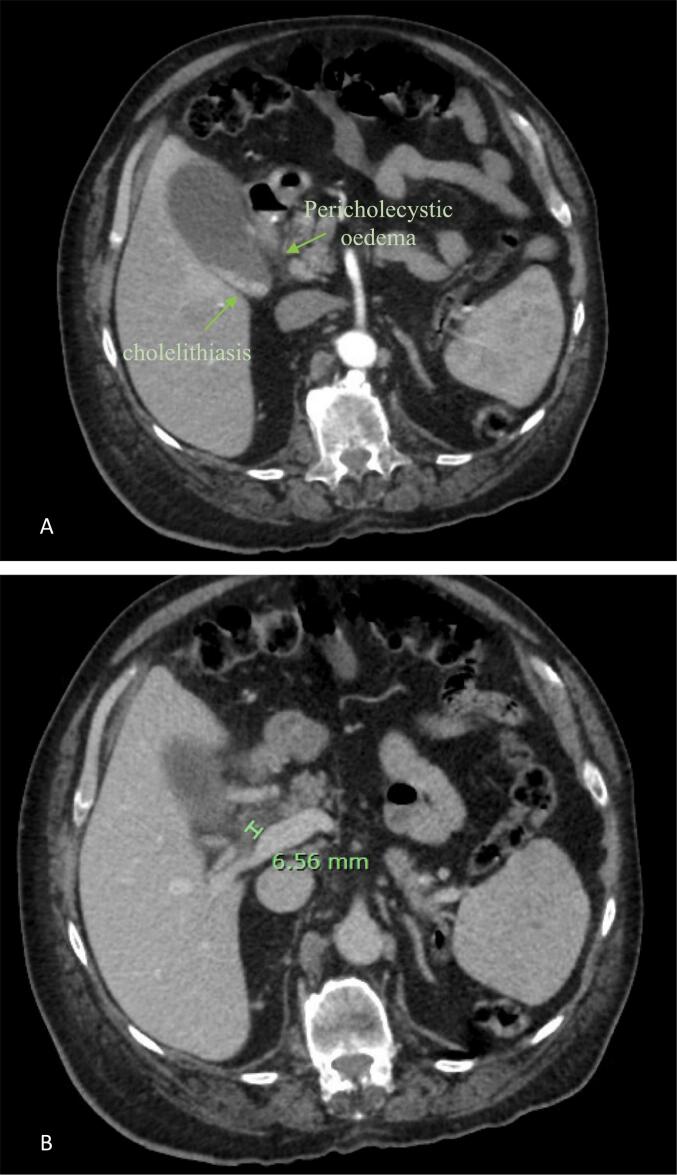
Axial section on Computed Tomography of Abdomen Pelvis (CTAP) showing cholelithiasis, peri-cholecystic oedema (A) and common bile duct diameter (B).

**Fig. 2:**
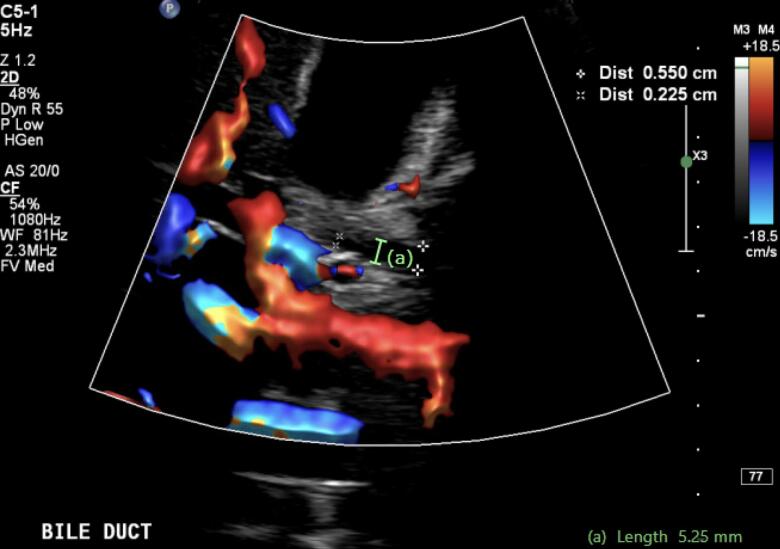
Initial ultrasound demonstrating non dilated proximal common bile duct. Limited view due to patient's body habitus.

A Computed tomography (CT) scan with Intravenous (IV) contrast was performed as part of the workup, which showed small calculi within gallbladder and evidence of pericholecystic oedema without gallbladder wall thickening (Fig. 1). There were no demonstrable choledocholithiasis or biliary or hepatic ductal dilation. A subsequent transabdominal biliary ultrasound demonstrated a small volume of sludge, mobile cholelithiasis and a non-dilated proximal common bile duct (CBD) with a diameter of 5.2 mm (Fig. 2). A magnetic resonance cholangiopancreatography (MRCP) was planned but did not proceed as the patient's abdominal girth exceeded the maximum state-wide machines' bore calibre despite full expiration.

Initial plans for either a laparoscopic cholecystectomy and bile duct exploration or ERCP were delayed due to patients admission being complicated by a Type 2 Non ST Elevation Myocardial Infarction (NSTEMI) and therefore deemed as high risk for general anaesthetic by the peri-operative and anaesthetic team. [3]. Fortunately, the patient clinically and biochemically improved with medical management of IV piperacillin and tazobactam, with bilirubin level improving to 15 from 55 (<20 μmol/L). On day eight he was discharged home with presumed passing of the choledocholithiasis, recommencement of his anticoagulation and scheduling a clinic follow up.

**Fig. 3:**
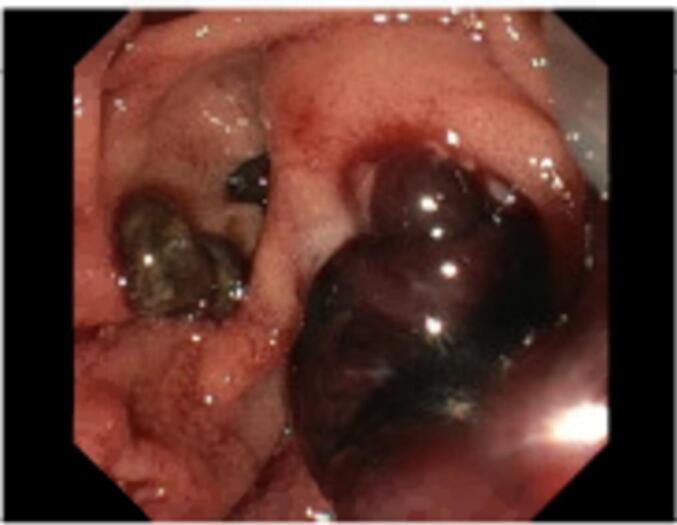
Endoscopic Retrograde Cholangiopancreatography (ERCP) image demonstrating clot at orifice pre-sphincterotomy.

**Fig. 4:**
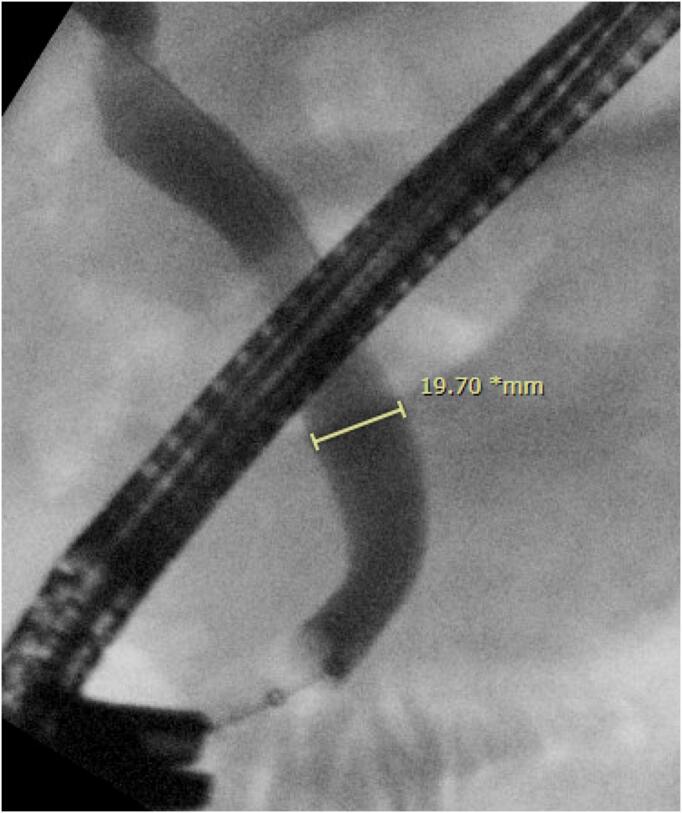
Intraoperative Cholangiogram (IOC) demonstrating dilated common bile duct.

The patient re-presented to hospital the following day with epigastric pain and worsening liver function test and hyperbilirubinaemia; bilirubin 56 (<20 μmol/L), ALP 331 (30–110 U/L), GGT 706 (<55 U/L) and CRP 35(<10 mg/L). His coagulation profile remained unremarkable. A repeat ultrasound did not demonstrate biliary dilatation on the visualised short segment CBD or evidence of obstruction. He was given IV piperacillin and tazobactam for presumed cholangitis and his apixaban was withheld for 3 days. He subsequently underwent an emergency ERCP which demonstrated a large mid to distal CBD clot obstructing the orifice with CBD dilatation to 12 mm (Fig. 3, Fig. 4). The discrepancy between imaging and ERCP findings was likely due to the patient's large body habitus causing suboptimal ultrasonographic visualisation of the CBD. A sphincterotomy was performed and the clot was retrieved. Post procedurally, the patient recovered well and was discharged home with a plan to restart apixaban 7 days post, as advised by the cardiology unit.

## 4 Discussion

Haemobilia or the presence of blood in the biliary tree, is a rare occurrence that is typically an iatrogenic complication of peri-biliary instrumentation [4]. Most non-iatrogenic cases are secondary to malignancies such as cholangiocarcinoma [5] and hepatocellular carcinoma with biliary ductal invasion [6,7]. Other causes include vascular malformations, trauma, abscesses and biliary fistula communicating with arterial structures [4]. Quincke's triad of right upper quadrant pain, jaundice and upper gastrointestinal bleeding presents in 25–30 % of patients [4,8,9]. CT angiogram is the proposed diagnostic tool of choice followed by upper endoscopy and ERCP [4] which aids in demonstrating attenuated clots, defining biliary fistulas [10] or pseudoaneurysms [11]. Management principles include coagulopathy correction and obstruction relief. Our case demonstrates the complexities that can arise in the haemobilia workup for some patients. This patient did not have the typical Quincke's triad of symptoms, instead he had generalised abdominal pain and absence of jaundice and melaena. Furthermore, his pre-procedural imaging of contrast enhanced CT was not optimal for defining the obstructive clot and biliary dilatation and has lower sensitivity compared to EUS or ERCP in detecting smalls stones or clots [12].

The patient's haemobilia was likely multifactorial; we hypothesise that the initial presentation was likely due to choledocholithiasis or cholangitis causing irritation to the ductal wall, then exacerbated by a transient vitamin K deficiency secondary to biliary obstruction with subsequent resumption of oral anticoagulation resulting in bleeding and clot formation. Abnormal coagulation profile to prove this hypothesis was not demonstrated as the patient had only two coagulation profiles performed due to delayed diagnosis and re-admission. There are few reported cases of spontaneous haemobilia in the literature. In a case series of 222 patients with haemobilia, only 4 or 2 % were caused by coagulopathy [8]; these were mostly on patients with bleeding disorders including haemophilia [13,14], Bernard-Soulier syndrome [15] and idiopathic thrombocytopenic purpura [16]. Very few researchers proposed it as a direct consequence of anticoagulation therapy [17,18]. Luzuy, Reinberg [19] reported two cases of chronic haemobilia that led to encrustation with bile constituents resulting in obstructive stones. Hiramatsu, Watanabe [17] proposed a similar mechanism, finding intraductal stones with atypically high bile content in patients on anticoagulation.

Management of clots causing obstructive jaundice depends on both the cause and clinical picture. This patient was a poor surgical candidate due to his high anaesthetic risk and fortunately had successful, albeit delayed, clot extraction via ERCP. In cases where ERCP is not feasible, a few methods of clot dissolution have been reported. A recent case report by Smith, Simpson [18] utilised thrombolytic therapy via a percutaneous cholecystostomy tube for a comorbid patient with obstructive clot in the gallbladder. Daily alteplase flushes were given with good clot resolution demonstrable on serial cholecystograms [18]. The management for blood clots originating from cystic artery pseudoaneurysms identified on CT angiogram is embolization [8,11]. In some cases, covered metal stents have also been used to relieve obstruction [20].

Resumption or cessation of patient's normal anticoagulation therapy may require multidisciplinary discussions to balance risk verse benefits. Our patient required resumption and continuation of apixaban as he had a high stroke risk with CHA_2_DS_2_VASc score of 4. We resumed anticoagulation accepting the risk of bleeding and potential clot recurrence. Most importantly, patient had sphincter instrumentation during his ERCP which mitigated the risk of future obstructive jaundice.

## 5 Conclusion

Early recognition and treatment of biliary sepsis is critical to prevent reversible clinical deterioration. Pre-procedure biliary imaging is useful for diagnosis and treatment; however, conventional workup may not be feasible for some patients. ERCP remains a useful diagnostic and therapeutic tool to achieve immediate source control. Our case demonstrates haemobilia as a cause of obstructive jaundice for a patient on anticoagulation.

## Author contribution

Iman Hameed: Data collection, interpretation, writing the paper

Yahya Al-Habbal: Conceptualisation, data analysis and interpretation

## Consent

Written informed consent was obtained from the patient for publication and any accompanying images. A copy of the written consent is available for review by the Editor-in-Chief of this journal on request.

## Ethical approval

Ethical approval was not required as per Western Health Research Ethics Committee as this is a case report and patient's details will be de-identified.

## Guarantor

Yahya Al- Habbal.

## Research registration number

Not applicable.

## Funding

No funding was acquired for the publication of this manuscript.

## Conflict of interest statement

There were no conflicts of interest in the production of this article.

## Data Availability

Clinic notes, radiology and pathology reports available at request.
